# Neurovisceral Syndrome in a Patient with Monoclonal Gammopathy of Undetermined Significance: A Confirmed Case of Variegate Porphyria

**DOI:** 10.7759/cureus.92741

**Published:** 2025-09-19

**Authors:** Iraj Fatima, Kashaf ad Duja Awais, Aasim Sehbai, Ali Awais Khan Tareen, Maheen Shaharyar

**Affiliations:** 1 Department of Medicine, Dubai Medical University, Dubai, ARE; 2 Department of Medicine, Rawalpindi Medical University, Rawalpindi, PAK; 3 Department of Hematology and Oncology, Alabama Cancer Care (ALCC), Anniston, USA; 4 Department of Hematology and Oncology, Regional Medical Center, Anniston, USA

**Keywords:** acute hepatic porphyria, autonomic nervous system dysfunction, monoclonal gammopathy of undetermined significance (mgus), neuropsychiatric symptom, neurovisceral attacks, peripheral neuropathy, variegate porphyria

## Abstract

Variegate porphyria (VP) is a rare disorder presenting with cutaneous, neurological, and systemic symptoms. We report a 53-year-old man initially hospitalized for a depressive episode, during which blistering lesions and facial flushing were noted. He was misdiagnosed and treated for cellulitis and contact dermatitis without improvement. Repeated misdiagnoses led to extensive autoimmune, infectious, and hematologic workup, revealing coexisting monoclonal gammopathy of undetermined significance (MGUS). Over the following months, he developed abdominal pain, mood swings, cognitive impairment, neuropathy, orthostatic symptoms, and weight loss, raising suspicion for porphyria. A 24-hour urine panel revealed elevated porphobilinogen (PBG), delta-aminolevulinic acid (ALA), coproporphyrin I and III, heptacarboxyl porphyrins, and mildly elevated uroporphyrins, confirming VP. Treatment with hemin caused thrombophlebitis and was discontinued. He was transitioned to Givosiran, resulting in symptom control and no further flares. This case reinforces the need to consider rare metabolic disorders in unexplained multisystem presentations and highlights the role of enzyme-targeting therapy when conventional treatment is not tolerated.

## Introduction

Porphyria refers to a group of inherited disorders resulting from defects in enzymes of the heme biosynthesis pathway. These defects result in the accumulation of toxic porphyrin precursors such as delta-aminolevulinic acid (ALA) and porphobilinogen (PBG), subsequently presenting as a constellation of symptoms across multiple systems [1].

Variegate porphyria (VP), an uncommon subtype of the acute hepatic porphyrias, is an autosomal dominant genetic metabolic disorder caused by mutations, resulting in partial deficiency of the enzyme protoporphyrinogen oxidase (PPOX). VP has a reported prevalence of one to nine per 1,000,000 individuals in the general population [2].

VP can display symptoms of both cutaneous porphyria, characterized by chronic skin blistering lesions, and acute hepatic porphyria, which manifests as a variety of complex neurovisceral symptoms. These episodes may involve intense abdominal pain, neuropathy, behavioural changes, vomiting, and autonomic disturbances such as tachycardia or orthostatic dysfunction [3]. This broad symptom range can make identifying a single underlying cause difficult and delay timely diagnosis. In such scenarios, it is important to distinguish between possible causative disease processes and include rare phenomena like porphyrias in the differential diagnosis to resolve uncertainties and initiate effective therapy.

An additional layer of complexity in this case was the coexistence of monoclonal gammopathy of undetermined significance (MGUS), a premalignant plasma cell disorder defined by the presence of a monoclonal protein without evidence of end-organ damage [4]. Although most patients with MGUS remain asymptomatic, some may develop neurological or dermatological manifestations, and recent literature has described these cases under the broader category of monoclonal gammopathy of clinical significance (MGCS) [5]. This overlap is clinically important, as it can obscure the presentation and complicate the diagnostic pathway in patients with suspected porphyria.

This case demonstrates how different diseases can mimic acute hepatic porphyrias and why an understanding of the neuropsychiatric and dermatological patterns of VP is necessary to reach an accurate diagnosis in complex situations.

## Case presentation

**Figure 1:**
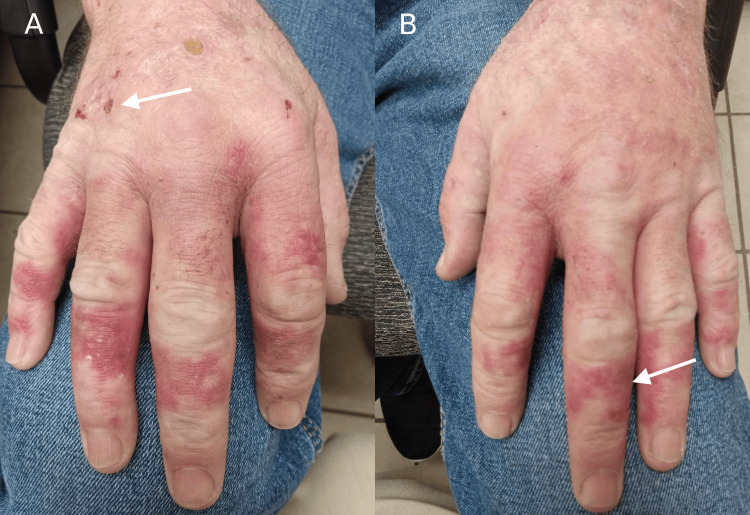
Dorsal hand involvement in variegate porphyria (VP). Panel A shows early blistering and crusting on the right dorsal hand (white arrow) in a sun-exposed distribution; panel B shows erythematous and violaceous lesions (white arrow) on the left hand.

**Figure 2:**
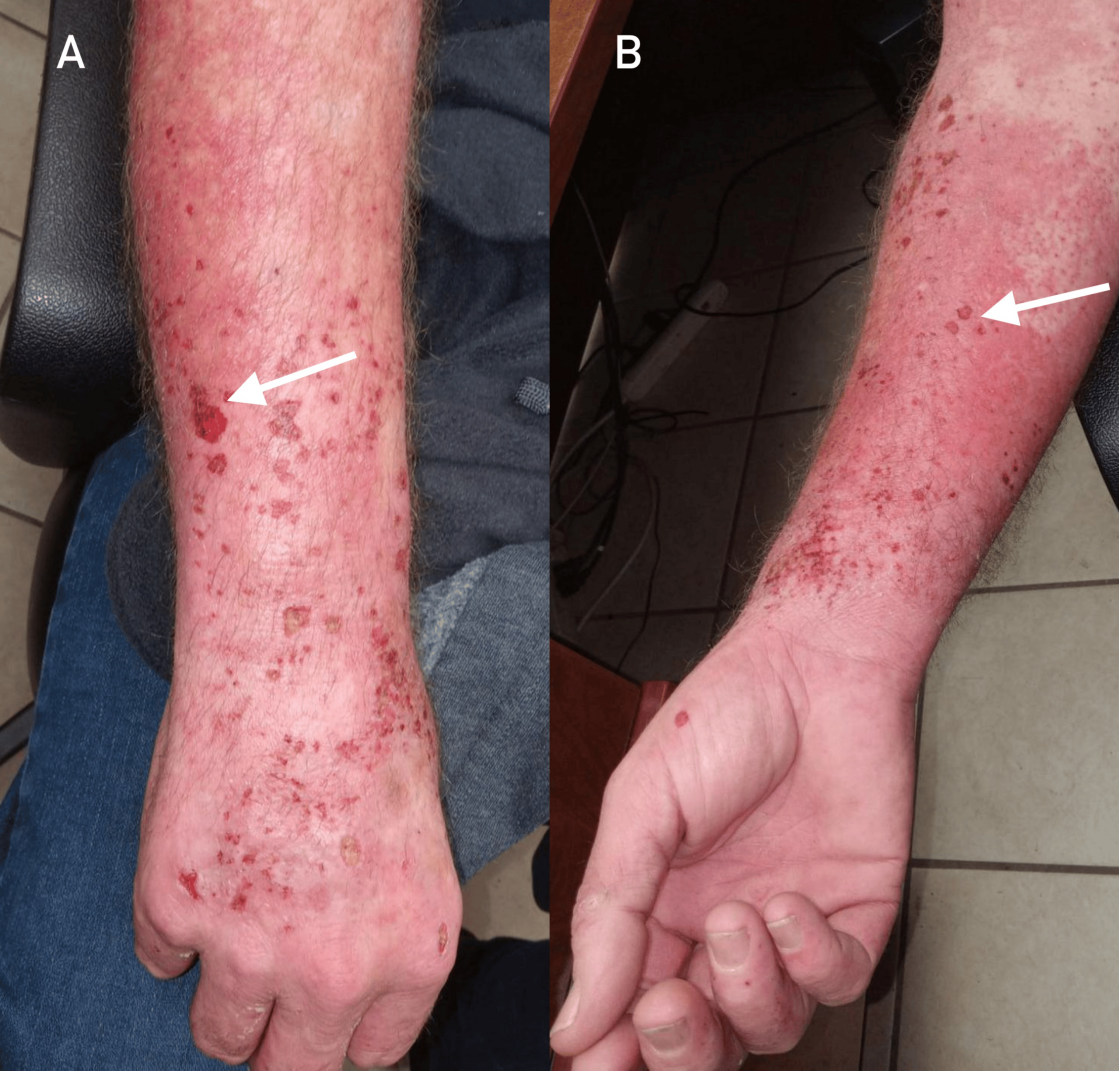
Acute photodermatitis flare in variegate porphyria (VP). Panel A shows the right forearm with erythema, erosions, and crusting (white arrow) over sun-exposed extensor skin. Panel B highlights the contralateral forearm, demonstrating similar blistering and superficial breakdown (white arrow).

A 53-year-old man was referred for evaluation of progressive skin and systemic symptoms. His past medical history included type 2 diabetes mellitus, hypertension, coronary artery disease, vitamin C deficiency, a family history of hepatocellular carcinoma, occasional alcohol consumption, and a longstanding smoking history. He initially presented with a severe attack of depression and anxiety requiring hospitalization for 10 days, during which he was noted to have painful, burning, and blistering skin lesions over the chest and arms (Figure 1), accompanied by facial flushing. He was started on oral antibiotics, which did not relieve any of his symptoms. Over the next three months, the patient’s condition deteriorated, and his cutaneous lesions worsened (Figure 2). He was subsequently referred to dermatology.

These cutaneous symptoms were initially misinterpreted as infectious cellulitis. He was hospitalised for suspected sepsis and treated with intravenous vancomycin and corticosteroids via midline access, without clinical improvement. Following a biopsy, he was diagnosed with contact dermatitis and prescribed topical corticosteroids, which also failed to relieve his symptoms. This warranted further workup for autoimmune and infectious diseases.

Multiple consultations were obtained with rheumatology and infectious diseases. The patient underwent a comprehensive workup including autoimmune serologies and blood cultures, all of which were unrevealing. His chronic cutaneous lesions, systemic symptoms such as abdominal pain and flushing, and the absence of autoimmune or infectious aetiology raised concern for a possible haematological disorder such as porphyria or a paraneoplastic process. The patient was subsequently referred for haematological evaluation.

A review of his laboratory results showed persistently elevated white cell counts dating back over a decade and an elevated serum chromogranin A. A positron emission tomography and computed tomography scan ruled out malignancy. Serum immunofixation revealed a monoclonal immunoglobulin G lambda spike. Bone marrow biopsy demonstrated normocellular marrow with 0.13% lambda-restricted plasma cells. Cytogenetic analysis by fluorescence in situ hybridisation detected low-level monosomy 13 and immunoglobulin heavy chain rearrangement, confirming the presence of MGUS.

Over the following three months, the patient experienced episodic symptoms of abdominal pain, pleurisy-like chest pain, mood instability, fatigue, tremors, and cognitive dysfunction described as “brain fog.” He also reported glove-distribution peripheral neuropathy and dizziness upon standing, suggestive of orthostatic hypotension, indicating possible autonomic nervous system dysfunction. Additional findings included progressive weight loss (approximately 4.5 kg).

Given this constellation of symptoms, a 24-hour urine panel was ordered at his next visit. The results showed raised levels of porphobilinogen (PBG), delta-aminolevulinic acid (ALA), coproporphyrin I and III, and heptacarboxyl porphyrins. Uroporphyrins were mildly elevated. These findings, summarized in Table 1, were diagnostic of an acute hepatic porphyria, particularly VP. 

**Table 1:** Investigations ranked by diagnostic relevance in VP with MGUS Abbreviations: PBG, porphobilinogen; ALA, delta-aminolevulinic acid; BMB, bone marrow biopsy; IgG, immunoglobulin G; SIFE, serum immunofixation electrophoresis; FISH, fluorescence in situ hybridisation; IGH, immunoglobulin heavy chain; VP, variegate porphyria; MGUS: monoclonal gammopathy of undetermined significance

Investigation	Result	Reference Range
PBG (24 h urine)	1.7 mg/24 h	0.0-1.5 mg/24 h
ALA (24 h urine)	3.3 mg/24 h	0.5-5.1 mg/24 h
Coproporphyrin III (24 h urine)	145 mcg/24 h	<90 mcg/24 h
Coproporphyrin I (24 h urine)	60 mcg/24 h	<30 mcg/24 h
Heptacarboxyl porphyrin (24 h urine)	5 mcg/24 h	<3 mcg/24 h
Uroporphyrins (24 h urine)	20 mcg/24 h	<15 mcg/24 h
Chromogranin A (serum)	139.3 ng/mL	<100 ng/mL
Bone marrow plasma cells (BMB)	0.13% lambda-restricted	<0.1%
Monoclonal IgG Lambda spike (SIFE)	Detected	Absent
FISH analysis (cytogenetic test)	Positive for monosomy 13 and IGH rearrangement	Negative

Differential diagnoses

Initial working diagnoses included infectious cellulitis, contact dermatitis, autoimmune vasculitis, and POEMS (Polyneuropathy, Organomegaly, Endocrinopathy, Monoclonal gammopathy, and Skin changes) syndrome. Infectious cellulitis was suspected due to the presence of blistering cutaneous lesions, but this was later excluded based on the absence of fever, negative blood cultures, and failure to improve with intravenous antibiotic therapy. A biopsy initially supported contact dermatitis; however, the lack of response to topical corticosteroids and the presence of systemic features made this diagnosis unlikely. Autoimmune vasculitis was considered due to skin involvement and concurrent systemic symptoms, but autoimmune serologies were negative and inflammatory markers were unremarkable. POEMS syndrome was also considered, given the presence of monoclonal gammopathy and peripheral neuropathy. However, the absence of organomegaly, endocrinopathy, or other defining features ruled it out. These exclusions led to consideration of acute hepatic porphyria. Although genetic testing is recommended for a definitive confirmation of VP, it was not performed in this patient due to resource limitations. Diagnosis was instead established based on clinical presentation and confirmatory biochemical testing.

Treatment

The patient was initially treated with intravenous hemin (Panhematin; Recordati Rare Diseases) administered daily for 10 days. However, therapy was discontinued due to the development of symptomatic thrombophlebitis at the infusion site, presenting with local erythema, tenderness, and induration along the vein. Supportive measures included discontinuation of infusion, warm compresses, and analgesics; no systemic complications occurred. No adjunctive therapies were provided at that time.

**Figure 3:**
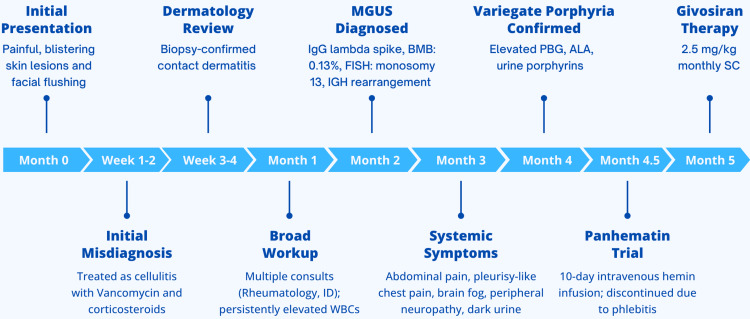
Timeline of clinical progression, investigations, and treatment in VP. Abbreviations: ALA, delta-aminolevulinic acid; BMB, bone marrow biopsy; FISH, fluorescence in situ hybridisation; IGH, immunoglobulin heavy chain; IgG, immunoglobulin G; MGUS, monoclonal gammopathy of undetermined significance; PBG, porphobilinogen; PPOX, protoporphyrinogen oxidase; SIFE, serum immunofixation electrophoresis; VP, variegate porphyria. This figure summarises the patient’s clinical course over 12 months, including initial cutaneous symptoms, diagnostic workup, MGUS confirmation, and therapeutic response to enzyme-targeted RNA interference therapy with Givosiran.

He was subsequently transitioned to subcutaneous Givosiran (Givlaari; Alnylam Pharmaceuticals, Cambridge, MA, USA) at a dose of 2.5 mg/kg administered once monthly. This transition occurred in the context of inadequate tolerance to hemin therapy (Figure 3).

The patient’s MGUS was managed conservatively with hematology follow-up, as there were no features to suggest progression to multiple myeloma or a related plasma cell disorder.

Outcome

At the time of writing, the patient has received three months of follow-up on Givosiran, with regular monthly hematology and dermatology reviews, including laboratory evaluation and symptom monitoring. During this period, he has experienced no recurrence of acute attacks, neurological symptoms, or cutaneous flares. He also reported significant improvement in his rash, neuropathic symptoms, cognitive function, and ability to perform activities of daily living. As VP is a chronic, relapsing condition, he remains under ongoing surveillance for disease activity. The patient is alive and clinically stable at the time of reporting.

## Discussion

VP is classified as a neurocutaneous porphyria because of its ability to cause photosensitive skin lesions and acute neurovisceral attacks. This atypical, multisystemic presentation often results in diagnostic uncertainty, as symptoms may resemble psychiatric, dermatologic, or gastrointestinal conditions, making it difficult to determine whether the underlying cause is malignant, autoimmune, or infectious [6]. The most common symptoms include abdominal pain, nausea, and vomiting, recurring in attacks and frequently preceding dermatologic flares. These cutaneous attacks typically manifest as severe photosensitivity, subepidermal blisters, and hyperpigmentation. Psychiatric disturbances, which can present as depression, anxiety, and mood disorders, are also common [7]. Autonomic dysfunction, while less frequent, is a recognised feature and may present as orthostatic hypotension, tachycardia, and constipation [8].

This case was unusual in several respects. The initial presentation with psychiatric symptoms, the co-existence of MGUS, and the early switch to Givosiran following intolerance to hemin collectively made diagnosis and management challenging. The patient first developed psychiatric and dermatologic symptoms without neurovisceral features, leading to initial misdiagnoses of cellulitis and contact dermatitis. This atypical presentation delayed recognition of acute hepatic porphyria. Ultimately, biochemical testing remains the cornerstone of diagnosis, with urinary PBG and ALA serving as first-line diagnostic tests during acute episodes [6]. In this patient, a 24-hour urine panel demonstrated elevated PBG, ALA, and heptacarboxyl porphyrins, confirming VP. Although genetic testing is recommended for definitive diagnosis and family screening [6,8], it was not performed in this case due to resource limitations.

The presence of MGUS further complicated the clinical picture. While MGUS is usually asymptomatic, it can occasionally present with neuropathy or dermatologic findings, which prompted a consideration of POEMS syndrome in this case [9]. The absence of organomegaly and endocrinopathy ruled out POEMS. In our patient, MGUS was most likely incidental, as no causal relationship between MGUS and VP has been established in the literature. Nevertheless, MGUS is increasingly recognized within the broader spectrum of monoclonal gammopathy of clinical significance (MGCS), where M-proteins may directly contribute to organ dysfunction. The reported manifestations most often involve the skin and peripheral nervous system [10]. Large cohort studies show that peripheral neuropathy is significantly more common in patients with MGUS than in the general population [11], and cutaneous signs such as purpura and other immunoglobulin-related rashes have also been reported [4]. These overlaps complicated the diagnostic process in our patient, since neuropathy, weight loss, and skin changes could plausibly be attributed to either VP or MGUS. Ultimately, biochemical testing confirmed VP as the unifying diagnosis, while MGUS served as an incidental but diagnostically distracting comorbidity.

Traditionally, VP is managed with glucose and intravenous hemin, as was initially attempted in this case [6]. However, the patient developed thrombophlebitis following hemin infusions, necessitating the use of an alternative agent. He was started on Givosiran, an RNA interference (RNAi) therapeutic approved in 2019. It is currently the only enzyme-targeting therapy that acts on hepatic delta-aminolevulinic acid synthase 1 (ALAS1), the rate-limiting enzyme in heme synthesis, thereby reducing the production of the neurotoxic precursors ALA and PBG [12]. Despite being a comparatively novel drug, Givosiran has demonstrated favorable safety and efficacy, with studies reporting a 74% mean reduction in porphyria attack rates and a 96% reduction in hemin use [13,14], resulting in improved quality of life in most patients. In this patient, monthly subcutaneous Givosiran at 2.5 mg/kg led to early symptom control, with no recurrence of neurovisceral or cutaneous flares during the initial three months of follow-up. While the duration of therapy is still short, the favorable early response is consistent with clinical trial data and supports the utility of enzyme-targeted therapy in patients intolerant of hemin [13,14].

Similar cases have described VP mimicking autoimmune, infectious, and hematologic disorders due to its systemic manifestations. Frank (2016), for instance, reported neuropsychiatric symptoms of VP initially misdiagnosed as psychiatric illness [15]. Barletta et al. (2021) published a systematic review highlighting the overlap between porphyrias and systemic conditions involving neuropathy and cutaneous lesions [7].

The current clinical guidelines from the European Porphyria Network (EPNET) and American Porphyria Foundation recommend that diagnosis of acute hepatic porphyrias, including VP, should begin with the measurement of urinary PBG and ALA during or shortly after an attack [6,8]. Confirmatory tests include plasma porphyrin scanning and genetic analysis when available [6,8]. Treatment includes carbohydrate loading or intravenous hemin, with Givosiran reserved for patients with frequent or hemin-intolerant attacks [13,14]. Long-term management focuses on trigger avoidance, sun protection, and regular follow-up with dermatology, neurology, and hematology [7-9].

## Conclusions

This case highlights the diagnostic uncertainty associated with VP, particularly when it co-exists with conditions like MGUS. While MGUS most likely represented an incidental comorbidity, its overlap with dermatologic and neurologic features contributed significantly to the diagnostic challenge, and no causal relationship between MGUS and VP has been demonstrated in the literature. It emphasises the importance of including acute hepatic porphyrias in the differential diagnosis, initiating early biochemical testing, and utilising a multidisciplinary approach in patients with vague, multisystemic symptoms. Furthermore, the case aligns with current clinical guidelines and supports the therapeutic role of RNAi therapy in severe or treatment-refractory presentations.
